# A Complex Case of Polymyositis Overlapping With Hypothyroid Myopathy Without Underlying Autoimmune Thyroid Disorder

**DOI:** 10.7759/cureus.8629

**Published:** 2020-06-15

**Authors:** Abhinav Garg, Michelle Helbig, Mark Schauer, Minh Nguyen

**Affiliations:** 1 Internal Medicine, Western Michigan University Homer Stryker M.D. School of Medicine, Kalamazoo, USA; 2 Internal Medicine/Pediatrics, Western Michigan University Homer Stryker M.D. School of Medicine, Kalamazoo, USA

**Keywords:** hypothyroidism, polymyositis, myopathy

## Abstract

A 78-year-old woman with a past medical history of hypothyroidism and Sjogren's syndrome presented with a two-month history of gradually progressive bilateral lower extremity weakness. Significant elevation in thyroid-stimulating hormone (TSH) and muscle enzyme, such as creatine kinase, was noticed on presentation. Due to concerns of hypothyroid myopathy, the patient was started on thyroxine and triiodothyronine supplementation. The patient reported no significant improvement in her weakness in the one-month follow-up. Laboratory workup revealed improving TSH levels but worsening creatine kinase levels. Electromyography study showed primarily myopathic features, such as abnormal insertional activity concerning for mild inflammatory myopathy. Muscle biopsy showed mild inflammatory exudate and features of myopathy with ongoing denervation. The patient was diagnosed with polymyositis and started on prednisone 0.5 mg/kg daily with a taper course and methotrexate. The patient reported significant improvement in her weakness when seen in six weeks with normalizing creatine kinase levels. The hallmark difference between hypothyroid myopathy (including polymyositis-like syndrome) and conventional polymyositis is the complete clinical recovery and resolution of laboratory abnormalities after treatment with thyroid hormone replacement in hypothyroid myopathy. There was no evidence of underlying autoimmune thyroid disorder which makes this case unique. This case highlights the complex case of polymyositis overlapping with hypothyroid myopathy with no underlying autoimmune disorder.

## Introduction

In addition to hypothyroidism, some common etiologies of myopathy include malignancy, hereditary causes, sarcoidosis, infection, electrolyte abnormalities, and certain drugs. Hypothyroid myopathy usually causes mild weakness, fatigue, and mildly elevated muscle enzymes. Patients with hypothyroid myopathy who do not respond to thyroid hormone replacement should be evaluated for other etiologies. Electromyography and muscle biopsy can be helpful in differentiating the underlying myopathy, though it can be non-specific in many cases.

## Case presentation

A 78-year-old woman with a past medical history of hypothyroidism, Sjogren’s syndrome, and atrial fibrillation presented to the clinic with a two-month history of gradually progressive bilateral lower extremity weakness causing difficulty climbing stairs. She also complained of arthralgias, myalgias, fatigue, and sandpaper sensation over her right anterior thigh. Symptoms of dry eyes and dry mouth from Sjogren’s syndrome were chronic and stable. Her hypothyroidism was controlled with the same dose of levothyroxine for over 10 years; normal thyroid-stimulating hormone (TSH) levels were noted eight months prior to presentation. Musculoskeletal examination showed neck flexor weakness, mild weakness in proximal upper extremities, and moderate weakness in proximal lower extremity muscle groups. Laboratory workup revealed normal electrolytes, elevated TSH of 204 U/mL, erythrocyte sedimentation rate of 89 mm/hour, antinuclear antibody ANA of 1:640, and negative anti-double-stranded DNA antibody. Her Sjogren's syndrome antibody (anti-SSA) levels were positive at 240 U/mL. Muscle enzymes were elevated, aldolase was 42 U/L and creatine kinase (CK) was 1,424 U/L. Due to concern for hypothyroidism-induced myopathy causing weakness and muscle damage, the patient was started on an increased dose of thyroxine and triiodothyronine supplementation. At the six-week follow-up, the patient reported no significant improvement in her leg weakness. Her TSH level had improved to 19 U/mL but her CK levels worsened to 2,895 U/L. The patient underwent electromyography study that showed primarily myopathic features, such as abnormal insertional activity concerning for mild inflammatory myopathy. Muscle biopsy of left vastus lateralis showed myopathic features, such as rare necrotic fibers replaced by macrophages, mild inflammatory exudate, muscle fiber variation, and necrosis. Mild grouping of type 1 fibers and an increase in non-specific esterase in atrophic fibers was noticed concerning for ongoing denervation. The clinical history, muscle biopsy findings, significantly elevated levels of muscle enzymes, and poor response to thyroid hormone supplementation pointed to the diagnosis of polymyositis. Her anti-JO 1 antibody for the anti-synthetase syndrome was negative. The patient was started on prednisone 0.5 mg/kg daily. At four-week follow-up, CK levels decreased to 1,910 U/L. The patient reported minimal improvement in her weakness. Methotrexate was added at 10 mg every week and prednisone increased to 60 mg/day with a six-week taper course. The patient was seen in six weeks and reported significant improvement in her symptoms. Her CK levels normalized 112 U/L. There was no evidence of autoimmune thyroid disorder (AITD) as anti-thyroglobulin antibody and anti-thyroid peroxidase antibody levels were negative. This case highlights an unusual presentation of polymyositis overlapping with hypothyroid myopathy.

## Discussion

Our patient presented with gradually progressive bilateral lower extremity weakness. The presence of systemic symptoms, such as easy fatigability and myalgias, along with the significantly elevated levels of TSH on presentation pointed towards hypothyroid myopathy. The patient’s muscle weakness did not improve with thyroid hormone replacement even after the TSH level normalized, which led to the search for other causes.

In addition to hypothyroidism, some common etiologies of myopathy include malignancy, hereditary causes, sarcoidosis, infection, electrolyte abnormalities, and certain drugs. Autoimmune disorders, such as polymyositis, dermatomyositis, inclusion body myositis, and overlap syndromes, also cause myopathy. These conditions should not be overlooked in a patient with suspected hypothyroid myopathy who does not improve with thyroid hormone replacement. The pathognomonic feature of hypothyroid myopathy is the clinical improvement of myopathy and resolution of laboratory abnormalities after thyroid hormone replacement [1]. Hypothyroidism causes myopathy by suppressing mitochondrial oxidative phosphorylation and glycogenolysis in skeletal muscle which inhibits the main oxidative pathways of energy production [2]. Electromyography in hypothyroid myopathy shows a decreased duration of motor unit potentials and an increased number of polyphasic potentials, but these findings occur in only half of the patients [3]. Muscle biopsy in hypothyroid myopathy can be normal in most cases. The most common changes noted on biopsy are type 1 fiber hypertrophy with selective atrophy of type 2 fibers. Type 2 fibers have an increased dependence on anaerobic glycolysis in comparison to type 1 fibers. Some of the cases reported findings of myofiber necrosis and the presence of core-like structures on biopsy [4]. These histopathology findings are similar to the findings seen in the course of denervation in neurogenic muscle diseases [5]. Our patient’s muscle biopsy showed mild grouping of type 1 fibers and an increase in non-specific esterase in atrophic fibers concerning for ongoing denervation. This led us to believe that our patient might have a component of hypothyroidism contributing to her muscle weakness in addition to polymyositis which is explained below.

A rare form of hypothyroid myopathy is a polymyositis-like syndrome in which the muscle enzymes are significantly elevated like those in inflammatory myopathies. This makes it difficult to differentiate between the two myopathies [6]. There is extensive involvement of axial and pelvic muscles in the polymyositis-like syndrome as compared to hypothyroid myopathy in which this is rare. There are no specific findings on electromyography and muscle biopsy that have been identified for this syndrome [7]. The hallmark for diagnosis of polymyositis-like syndrome in hypothyroidism is the clinical response to thyroid hormone replacement similar to that seen in hypothyroid myopathy. This helps to differentiate this entity from polymyositis, which does not respond to thyroid hormone replacement [8].

Polymyositis is a chronic autoimmune disorder involving progressive proximal muscle weakness and significantly elevated muscle enzymes. Association of polymyositis with other autoimmune disorders, such as mixed connective tissue disease (MCTD), systemic lupus erythematosus (SLE), scleroderma, and Sjogren’s syndrome, is well known. But there are few reports on the association of polymyositis with an AITD. Systemic and thyroid autoimmune disorders can often overlap. As compared to Graves’ disease, the coexistence of Hashimoto’s thyroiditis was found to be more common in patients with systemic autoimmune diseases [9]. The coexistence of polymyositis with AITD can be explained by shared environmental and genetic factors [10]. There was no evidence of AITD in our patient which makes this case unique. Patients with polymyositis with a positive anti-JO 1 antibody have a higher chance of lung involvement and development of interstitial lung disease. Polymyositis diagnosis can often precede the diagnosis of hypothyroidism; it is usually diagnosed later in patients with hyperthyroidism. Poor prognosis in polymyositis is associated with the presence of malignancy, older age, and presence of anti-SSA antibodies [11]. Our patient is an elderly female with a positive history of Sjogren’s syndrome, and her anti-SSA antibody levels were elevated. Malignancy is more common in polymyositis patients with underlying hypothyroidism than hyperthyroidism [12]. Ovarian and lung cancer are the most common associated cancers with polymyositis [13].

Electromyography in polymyositis can show polyphasic motor unit potentials of low amplitude and short duration along with fibrillation waves and insertional irritability [14]. Muscle biopsy can show non-specific features of myopathy, such as variation of fiber size, along with foci of degeneration, necrosis, and extensive mononuclear inflammatory infiltrate [15]. The electromyography in our patient showed primarily myopathic features, such as abnormal insertional activity. The presence of inflammatory exudate around endomysial blood vessels, muscle fiber variation, and necrosis in our patient was compatible with polymyositis.

## Conclusions

Both hypothyroidism and polymyositis can present as muscle weakness with elevation in serum muscle enzyme levels. Hypothyroid myopathy usually causes mild weakness, fatigue, and mildly elevated muscle enzymes. It can also present with significantly elevated levels of muscle enzymes and a polymyositis-like syndrome. The hallmark difference between hypothyroid myopathy (including polymyositis-like syndrome) and conventional polymyositis is the complete clinical recovery and resolution of laboratory abnormalities after treatment with thyroid hormone replacement in hypothyroid myopathy. Polymyositis, however, requires immunosuppressive therapy. Patients with apparent hypothyroid myopathy who do not respond to thyroid hormone replacement should be evaluated for other etiologies. Electromyography and muscle biopsy can be helpful in differentiating the underlying myopathy, though it can be non-specific in many cases. Polymyositis when associated with malignancy, older females, and positive anti-SSA antibody has a poorer prognosis. 

## References

[1] Cabili S, Kaplinsky N, Pines A, Frankl O (1982). Hypothyroidism masquerading as polymyositis. Postgrad Med J.

[2] McDaniel HG, Pittman CS, Oh SJ, DiMauro S (1977). Carbohydrate metabolism in hypothyroid myopathy. Metabolism.

[3] Sindoni A, Rodolico C, Pappalardo MA, Portaro S, Benvenga S (2016). Hypothyroid myopathy: a peculiar clinical presentation of thyroid failure. Review of the literature. Rev Endocr Metab Disord.

[4] Mastaglia FL, Ojeda VJ, Sarnat HB, Kakulas BA (1988). Myopathies associated with hypothyroidism: a review based upon 13 cases. Aust N Z J Med.

[5] Vita G, Migliorato A, Baradello A (1994). Expression of cytoskeleton proteins in central core disease. J Neurol Sci.

[6] Aslam H, Sayeed MA, Qadeer R, Afsar S (2015). Hypothyroidism simulating as polymyositis. J Pak Med Assoc.

[7] Madariaga MG, Gamarra N, Dempsey S, Barsano CP (2002). Polymyositis-like syndrome in hypothyroidism: review of cases reported over the past twenty-five years. Thyroid.

[8] Yüksel G, Akpinar A, Çömez N, Örken C, Tireli H (2007). Hypothyroidism presenting as a polymyositis-like syndrome (case report). J Neurol Sci.

[9] Biró E, Szekanecz Z, Dankó K (2006). Association of systemic and thyroid autoimmune diseases. Clin Rheumatol.

[10] Szyper-Kravitz M, Marai I, Shoenfeld Y (2005). Coexistence of thyroid autoimmunity with other autoimmune diseases: friend or foe? Additional aspects on the mosaic of autoimmunity. Autoimmunity.

[11] Airio A, Kautiainen H, Hakala M (2006). Prognosis and mortality of polymyositis and dermatomyositis patients. Clin Rheumatol.

[12] Yazici Y, Kagen LJ (2000). The association of malignancy with myositis. Curr Opin Rheumatol.

[13] Smith CJ, Staniland JR (2003). A difficult case of inflammatory myositis. Age Ageing.

[14] Wang H, Li H, Kai C, Deng J (2011). Polymyositis associated with hypothyroidism or hyperthyroidism: two cases and review of the literature. Clin Rheumatol.

[15] Bohan A, Peter JB, Bowman RL, Pearson CM (1977). A computer-assisted analysis of 153 patients with polymyositis and dermatomyositis. Medicine.

